# Cytokine Directed Chondroblast Trans-Differentiation: *JAK* Inhibition Facilitates Direct Reprogramming of Fibroblasts to Chondroblasts

**DOI:** 10.3390/cells9010191

**Published:** 2020-01-11

**Authors:** Perla Cota, Summer A. Helmi, Charlie Hsu, Derrick E. Rancourt

**Affiliations:** 1 Department of Biochemistry and Molecular Biology, University of Calgary, 3330 Hospital Dr. NW, Calgary, AB T2N 1N4, Canada; perla.cota@helmholtz-muenchen.de (P.C.); Summer.helmi@ucalgary.ca (S.A.H.); c.hsu@uq.net.au (C.H.); 2 Institute of Diabetes and Regeneration Research, Helmholtz Zentrum München, German Research Center for Health and Environment, 85764 Neuherberg, Germany; 3 Department of Oral Biology, Faculty of Dentistry, Mansoura University, Mansoura 35516, Egypt; 4 Faculty of Medicine University of Queensland. 20 Weightman St, Herston 4006, QLD, Australia

**Keywords:** trans-differentiation, osteoarthritis, cartilage regeneration, cellular reprogramming

## Abstract

Osteoarthritis (OA) is a degenerative disease of the hyaline articular cartilage. This disease is progressive and may lead to disability. Researchers proposed many regenerative approaches to treat osteoarthritis, including stem cells. Trans-differentiation of a fully differentiated cell state directly into another different differentiated cell state avoids the disadvantages of fully reprogramming cells to induced pluripotent stem cells (iPSCs) in terms of faster reprogramming of the needed cells. Trans-differentiation also reduces the risk of tumor formation by avoiding the iPSC state. *OSKM* factors (*Oct4, Sox2, Klf4, and cMyc*) accompanied by the JAK-STAT pathway inhibition, followed by the introduction of specific differentiation factors, directly reprogrammed mouse embryonic fibroblasts to chondroblasts. Our results showed the absence of intermediate induced pluripotent stem cell formation. The resulting aggregates showed clear hyaline and hypertrophic cartilage. Tumor formation was absent in sub-cutaneous capsules transplanted in SCID mice.

## 1. Introduction

Osteoarthritis (OA) is a consequence of the degeneration of hyaline articular cartilage, which leads to the formation of fibrocartilage. Fibrocartilage has different biomechanical properties compared to the hyaline cartilage present in healthy joints and does not protect subchondral bone following degeneration [1]. Current cell therapies include using chondroblasts obtained from existing cartilage tissue [2]. The most common approach is microfracture chondroplasty, which uses penetration of the subchondral bone to deliver non-chondroblast endogenous progenitor cells from the bone marrow into the defected area. Another method is autogenous osteochondral transplantation/mosaicplasty, which transfers autologous osteochondral grafts to the affected area. Lastly, there is autologous chondroblast implantation performed after isolating chondroblasts from a piece of the cartilage of a small non-load bearing area of the knee joint. These cells are then expanded in vitro and transplanted into the chondral defect [3].

Since there are few treatments for osteoarthritis, the main options to alleviate the symptoms are physical therapy, weight loss, non-steroidal anti-inflammatory drugs (NSAIDs), injections of hyaluronic acid (HA), and, lastly, total joint replacement at end-stage osteoarthritis [4]. Physical activity proved to play an important role in the treatment of osteoarthritis [5]. This study also showed that diet modification could be an essential factor in the recruitment of the progenitor cell pool into the chondrocyte fate at the injury site. Autologous stem cell transplants can offer a near future therapy for osteoarthritis. Mesenchymal stem cells derived from bone marrow or adipose tissue offer one potential approach for repairing cartilage tissue [6]. However, law-scale harvesting and replicative senescence limit the full utility of this approach [7,8]. Alternatively, patient-derived induced pluripotent stem cells (iPSCs) are an abundant source of stem cells, which can be used to derive cartilage tissue [9,10]. However, one concern is the tumorigenic capacity of iPSC-derived tissue, which can occur during chondrogenic differentiation [9]. In this case, we propose that trans-differentiation of patient fibroblasts to chondroblasts provides a shorter path for cartilage repair.

The first trans-differentiation attempt was achieved long before the discovery of iPSCs. The myogenic determination gene (*MyoD*) changed the fate of fibroblasts to skeletal muscle cells [11]. *MyoD*’s discovery designated that overexpression of a key transcription factor was sufficient to alter and abolish the endogenous gene expression pattern of a cell. Multiple studies demonstrated that it is possible to directly trans-differentiate one adult cell type to the other using small molecules. Other studies investigated the underlying mechanisms [12,13,14]. These experiments, however, demonstrated that changes could only take place within cells from the same germ layer. In these cases, this includes mesoderm and ectoderm layers [15].

The derivation of iPSCs demonstrated that transcription factors could completely reverse cellular fate to the embryonic stem cell (ESC) state. Whereas *Oct4* and *Sox2* play essential roles in establishing and maintaining the ESC state, *Klf-4* and *c-Myc* were instrumental in opening chromatin, which enables *Oct4* and *Sox2* to act [16]. In the stochastic model of cellular reprogramming [17], Yamanaka predicted that his cellular reprogramming approach directed cells to move from one fate to another via partial reprogramming, which is also known as trans-differentiation.

Since then, many transcription factor combinations were successful in the trans-differentiation of one cell type to the other. Examples would include the use of 13 transcription factors responsible for cardiac differentiation. These transcription factors were narrowed down to combining *Gata4*, *MeF2c*, and *Tbx5.* This combination was sufficient to reprogram mouse dermal fibroblasts directly to differentiated cardiomyocyte-like cells [18]. In a similar experiment, 19 transcription factors successfully handled neuron differentiation and were narrowed down to combining *Ascl1*, *Brn2*, and *Myt11* to convert mouse fibroblasts into functional neurons [19]. This technique successfully generated osteoblasts. *Oct4*, *L-Myc*, *Runx2*, and *Osterix* drastically induced fibroblasts to produce calcified bone matrix and express osteoblast-specific markers [20]. A study reported the generation of chondrogenic cells expressing no type I collagen through the transduction of *c-Myc, Klf4*, and *SOX9* [21]. Screening for transcription factors to induce trans-differentiation proved to be time-consuming. Furthermore, the produced cell type has a minimal capacity to divide and expand, which makes it challenging to be used clinically for the correction of tissue defects.

An alternative trans-differentiation method uses a combination of partial reprogramming combined with directed differentiation. In addition to removing the pluripotency maintenance factor leukemia inhibitory factor (*LIF*), this method blocks complete reprogramming using the JAK inhibitor JI1 [22]. Subsequently, chemicals and growth factors redirect differentiation toward the cell lineage of interest. This approach generated cardiomyocytes and neural tissue [22]. Moreover, human smooth muscle cells were trans-differentiated to endothelial cells using transient transduction with the Yamanaka factors [23].

In this study, we were successful in the trans-differentiation of mouse embryonic fibroblasts (MEFs) to chondroblasts. Retroviral transduction of MEFs using the traditional OSKM factors (*Oct4, Sox2, Klf4, and cMyc*) initiated the reprogramming process. Partial reprogramming of the cells took place through the removal of *LIF* in addition to JAK-STAT pathway inhibition. This reprogramming method placed the chromatin into a plastic state that allowed chemicals to redirect differentiation toward chondroblast. The produced chondroblasts were able to generate a normal cartilage matrix. The produced chondroblasts showed no tendency to form tumors upon in vivo transplantation.

## 2. Materials and Methods

### 2.1. Mouse Embryonic Fibroblast (MEFs) Isolation

Mouse embryonic fibroblasts (MEFs) were isolated from E13.5 embryos provided by Centre for Mouse Genomics, Cumming School of Medicine, University of Calgary. After removing the head, viscera, and spinal column, embryos were incubated in cold 0.05% trypsin (4 °C, Invitrogen, Burlington, ON, Canada) overnight. Embryos were then transferred to warm 0.25% trypsin (37 °C, Invitrogen) for 10 min and then triturated to obtain a single-cell suspension. Trypsin was inactivated with Dulbecco’s modified Eagle medium (DMEM) supplemented with 10% fetal bovine serum (FBS) (Invitrogen), and 50 U of penicillin-streptomycin (Invitrogen). Dissociated MEFs were then plated and expanded.

Genetically modified MEFs were isolated from B6 and CBA-Tg (*Pou5f1-EGFP*)2Mnn/J mice using the same isolation technique. The transgene contains an Enhanced Green Fluorescent Protein gene under the control of the promoter and distal enhancer of the POU domain, class 5, transcription factor 1 (*Pou5fl* or *Oct4*).

### 2.2. OSKM Retrovirus Packaging

Platinum-E (Plat-E) retroviral packaging cells (Cell Biolabs, San Diego, CA, USA) were prepared for plasmid transfections by seeding 8 × 10^6^ per 100mm dish (one dish for each reprogramming gene). Plat-E cells were maintained in Fibroblast-Platinum (FP) medium (Dulbecco’s modified Eagle medium (DMEM), 10% FBS, and 50 U of penicillin-streptomycin). After 24 h, we introduced each pMXs retroviral plasmid DNA (*Oct4, Sox2, Klf4, c-Myc,* and *Ds-Red*), (Addgene plasmids 13366, 13367, 13370, 13375 and 22724, respectively) into Plat-E cells using X-tremeGENE 9 DNA transfection reagent (Roche, High River, AB, Canada), according to the manufacturer’s recommendations. Furthermore, 18 μL of X-tremeGENE 9 transfection reagent was added to 300 μL of OptiMEM to a 1.5-mL tube. A total of 8 μg of each retroviral vector was added into the prepared XtremeGENE9-OptiMEM tube drop-by-drop and incubated for 15 min. Each vector–XtremeGENE 9 complex was added dropwise into the Plat-E cell–containing dishes and incubated overnight at 37 °C, 5% CO_2_. The following day, the medium was replaced with 10 mL of fresh FP medium. Forty-eight hours after transduction, we collected virus-containing medium from each transfection by filtering through a 0.45 μm Acrodisc filter (Pall Life Sciences, Mississauga, ON, Canada).

### 2.3. OSKM Retroviral Transductions

MEFs were initially passaged in Dulbecco’s modified Eagle medium (DMEM) supplemented with 10% FBS and 50 U of penicillin-streptomycin. Cells were seeded onto six-well plates overnight at 37 °C, 5% CO_2_ at 3.5 × 10^4^ cells per well in the previous media. The next day, equal parts of retroviral-conditioned medium supplemented with 0.5 μg/mL polybrene were mixed and added drop-by-drop to the prepared MEFs.

### 2.4. Cellular Reprogramming

Twenty-four hours after transduction, cells were washed with PBS and switched to Reprogramming Medium 1 (Knockout DMEM with 5% Knockout serum replacement, 15% ES cell-qualified FBS, 1% Glutamax, 1% non-essential amino acids, and 0.1 mM β-mercaptoethanol. All components from Invitrogen) for the first 6 days. After day 6, media was changed to Reprogramming Medium 2 (Knockout DMEM, 1% FBS, 14% KSR, 1% Glutamax, 1% nonessential amino acids, and 0.1% mM mercaptoethanol). During the first 9 days, JI1 (0.5 μM Millipore, Etobicoke, ON, Canada) was added. Fresh medium was added every 48 h throughout the experiment.

### 2.5. Chondroblast Differentiation

On day 10, cells were dissociated using 0.1% trypsin-EDTA (Invitrogen). Dissociated pre-iPSCs were cultured at a high-density, 1.0 × 10^5^ cells per 10 μL × 9 spots in 6 cm culture dish for 2 h. After incubation, the medium was added to all dishes without dissociating the cell drops. For chondroblast differentiation, we used differentiation media containing DMEM (Gibco, Burlington, ON, Canada), 1% non-essential amino acids (Invitrogen), 50 U/mL penicillin and 50 μg/mL streptomycin (Invitrogen), 0.1 mM 2-mercaptoethanol (Invitrogen), 1% ITS (Invitrogen), 1% FBS (Gibco), 10 ng/mL TGF-β1 (PeproTech, Rocky Hill, NJ, USA), 10 ng/mL BMP-2 (PeproTech), and 50 μg/mL ascorbic acid (Sigma, High River, AB, Canada). The medium was changed every 2 days. After 5 days, resulting pre-iPSC aggregates were separated from the dishes by pipetting. The separated aggregates were transferred to suspension culture in Petri dishes containing differentiation media, DMEM, 1% non-essential amino acids, penicillin-streptomycin, 0.1 mM 2-mercaptoethanol, 1% ITS, 1% FBS, 10 ng/mL BMP-2, and 50 μg/mL ascorbic acid.

### 2.6. RNA Isolation

Total RNA was isolated from cell cultures at different time points (days 0, 2, 6, 9, 12, 15, and 36) using an RNeasy Mini kit (Qiagen Toronto, ON, Canada) according to the manufacturer’s instructions with on-column DNase I digestion. RNA was measured using a NanoPhotometer P-Class (IMPLEN). In this case, 0.5–1 μg of total RNA was used as a template for cDNA synthesis with the High Capacity cDNA Archive Kit (Applied Biosystems, Calgary, AB, Canada).

### 2.7. Quantitative PCR

Quantitative polymerase chain reaction (Q-PCR) was performed using a Fast SYBR green gene expression master mix (Applied Biosystems) with 50 nM primer concentration. The cycles used were those recommended for the Fast SYBR green reagent by Applied Biosystems. The cycles included 20 s at 95 °C as an initial denaturation, which is followed by 3 s at 95 °C and then 30 s at 60 °C for annealing. Then it was 15 s of extension at 95 °C for 40 cycles. Mouse pluripotency markers include *Nanog* and *Rex-1*, and chondrogenic markers include *Sox9*, *Col2, Col10*, and *Mmp13*. Gene expression was quantified during a time course of maintenance using the ΔCt method. Relative gene expression was normalized to *Rsp29* and compared to day 0 MEFs and chondroblasts.

### 2.8. Flow Cytometry

MEFs and MEFs undergoing trans-differentiation were subjected to flow cytometry analysis under similar staining conditions. Both were dissociated with 0.25% trypsin for 5 min at 37 °C. Cells were then washed two times with PBS (Gibco) using centrifugation 126× *g* for 5 min each time. MEFs undergoing trans-differentiation were later permeabilized with 0.5% Triton X-100 (Sigma T8532) in PBS for 10 min at room temperature. After washing twice with PBS, the cells were treated with a blocking buffer (1% BSA, 0.1% Triton X-100 in PBS for MEFs, and 10% bovine serum albumin (BSA), 0.1% Triton X-100 in PBS) for 1 h. The primary antibody (anti-*Oct4*) was diluted in the blocking solution (1:100) and incubated for 1 h at room temperature. Cells were washed with 2 mL of PBS twice. Secondary antibodies were also diluted in the blocking solutions (1:1000) and incubated with the cells for 1 h at room temperature.

For MEFs, conjugated antibodies (anti-*H2kk* and anti-*Col2*) were diluted in flow cytometer buffer (1:100) and incubated for 1 h at room temperature. Lastly, the cells (MEFs and MEFs undergoing trans-differentiation) were washed with 2 mL of PBS twice, and a final fixation was performed with 3.7% Paraformaldehyde (PFA) for 30–45 min at room temperature. Lastly, PFA was diluted with PBS (Gibco) to a final volume of 5 mL. These cells were later centrifuged twice at 126× *g* for 5 min to remove all residue of PFA. An Attune Flow Cytometer with a 488 nm and 633 nm laser diode detected MEFs labeled for *Col2* and *H2kK*. MEFs that have a transgenic modification with a GFP gene insertion driven by an *Oct4* promoter were used to identify GFP positive cells after transduction at different time points (2, 6, and 9 days) by an Attune Flow Cytometer equipped with a 488 nm laser. Additionally, the presence of exogenous *Oct4* was detected after being labeled with the anti-*Oct4* antibody and was recorded under the same conditions.

### 2.9. Transplantation

Severe combined immune deficient (SCID) mice were ordered from Taconic company (Rensselaer, NY, USA) and housed in the animal facility of the Faculty of Medicine, University of Calgary. Technician Shiying Liu performed experiments as approved by the University of Calgary Health Sciences Animal Care Committee (Protocol AC15-0124). Cell aggregates were taken from static cultures after four weeks of trans-differentiation. Two mice were injected with trans-differentiated cells in a total volume of 200 μL PBS into a skin fold of the inner thigh. On day 21, the mice were sacrificed, and tissues were dissected and analyzed by histological procedures.

### 2.10. Histology

Aggregates at day 36 and isolated tissue from the transplantations were fixed with 4% paraformaldehyde (PFA) overnight at 4 °C. After dehydration, by increasing concentrations of ethanol, the aggregates and tissues were embedded in paraffin. Aggregates and tissue were sectioned and mounted. Next, the paraffin was removed from the slide that contained the aggregates and the tissues by rehydrating with diminishing concentrations of ethanol.

All the tissue sections were then stained with hematoxylin and eosin (H & E) and examined for cartilage tissue by light microscopy. Half of the aggregate sections were stained with H & E and the other half with Alcian blue (Sigma-Aldrich). Histology analysis was performed blinded.

### 2.11. Statistical Analysis

A two-sided unpaired student’s *t*-test was used to compare sample groups. Asterisks depict the following α levels: * *p* < 0.050, ** *p* < 0.010, *** *p* < 0.001, and n.s. *p* > 0.050 as not being significant.

## 3. Results

### 3.1. Evaluation of Transduction Efficiency and the Absence of Intermediate iPSCs Formation

**Figure 1:**
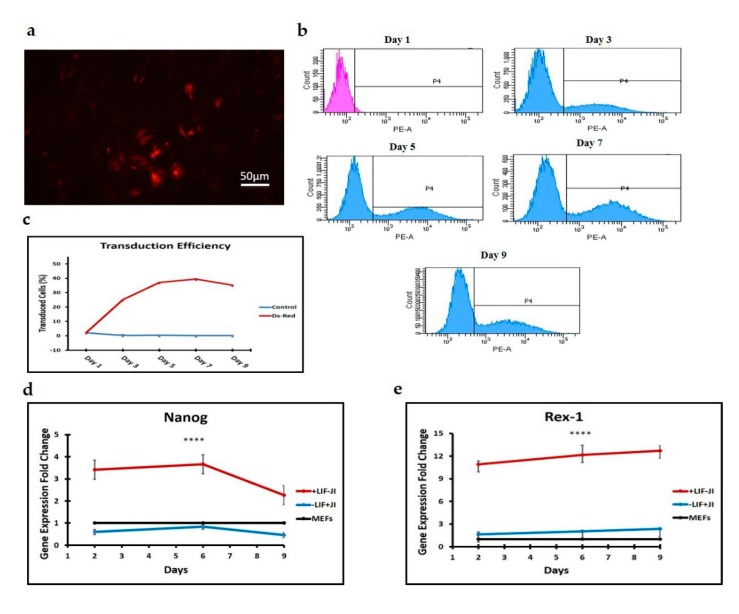
Transduction efficiency of MEFs using lentiviral vectors. To evaluate the efficiency of transduction, we utilized the fluorescence reporter gene *Ds-Red* and transduced MEFs as outlined in the materials and methods. (**a**) Transduced MEFs are fluorescent red under the microscope. Scale bar 50 μm. (**b**,**c**) Transgene expression reached a maximum on day 7 and maintained the signal up to day 9. (**d**,**e**) Upregulation of pluripotency gene expression in the presence of iPSC-inductive media (+LIF/−JI1), and trans-differentiation media (−LIF/+JI1). *Nanog* and *Rex-1* are significantly upregulated 3-fold and 10-fold, respectively, in cells under iPSC reprogramming media. **** *p* < 0.0001.

To evaluate the efficiency of transduction, we utilized the fluorescence reporter gene *Ds-Red* and transduced MEFs as outlined in the materials and methods. Overall, we achieved a 40% transduction efficiency. Transgene expression reached a maximum on day 7 and maintained the signal up to day 9 (Figure 1). 

Intermediate iPSCs formation during the trans-differentiation process was ruled out through the analysis of pluripotency marker gene expression. The gene expression fold change for the pluripotency markers *Nanog* and *Rex-1* was analyzed relative to MEFs. The analysis was performed in pluripotency reprogramming conditions (with LIF and absence of JI1) compared to the trans-differentiation reprogramming conditions (without LIF and with the addition of JI1) at days 2, 6, and 9 (Figure 1d, e).

Cells under iPSCs reprogramming conditions showed an overall higher expression of *Nanog* compared to the trans-differentiation reprogramming conditions, with significant upregulation (*p* = 0.0001) on day 6 (Figure 1d). Similarly, *Rex1* was highly expressed at every time point under the iPSC condition and significantly (*p* = 0.0001) higher on day 6 when compared to the trans-differentiation group (−LIF+JI1) (Figure 1e). These results indicated that LIF removal, combined with the JI1 addition, avoided intermediate iPSC formation.

**Figure 2:**
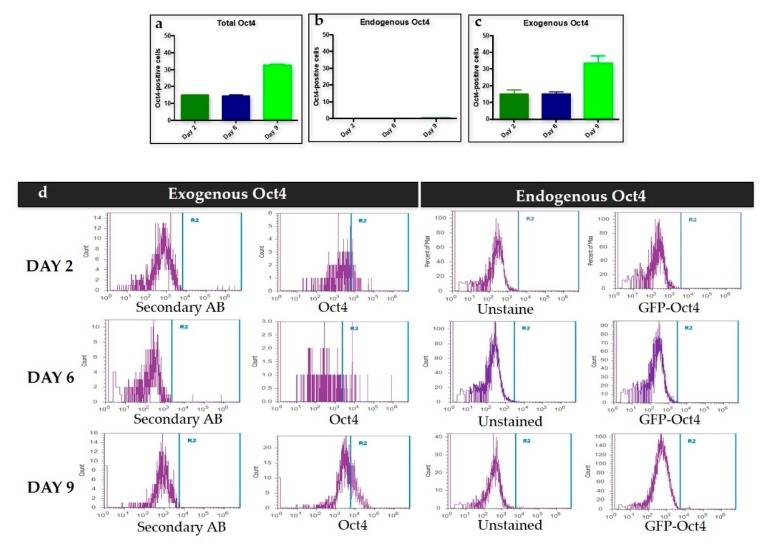
Exogenous, but not endogenous Oct4 expression during the first stage of trans- differentiation. The figure shows (**a**) the total signal of *Oct4* (exogenous and endogenous) at every time point, and (**b**) no endogenous *Oct4* at any time point. (**c**) Exogenous Oct4 signal was present since day 2 with the highest signal shown at day 9 indicating that partial reprogramming is occurring. (**d**) Histogram demonstrating the log change in exogenous *Oct4* compared to the absence of log change in endogenous *Oct4*. The absence of log change on endogenous *Oct4* and the log change on exogenous *Oct4* demonstrated the absence of iPSCs formation.

To confirm these results, we performed exogenous *Oct4* assessment to determine the potential formation of iPSC intermediates (Figure 2). We correlated the levels of endogenous and exogenous *Oct4* expression. MEFs used for trans-differentiation were isolated fromBL6 129S4-Pou5f1tm2Jae/J embryos, where the GFP gene expressed upon the activation of the *Oct4* promoter. MEFs from this strain would fluoresce green if endogenous *Oct4* was expressed [24]. Exogenous *Oct4* expression was measured by staining cells with a monoclonal antibody that targeted *Oct4* (Figure 2a–2c). Flow cytometry measured the number of positive cells for endogenous and exogenous *Oct4* of the trans-differentiated MEFs on days 2, 6, and 9. There was no signal detection for the expression of endogenous *Oct4* on days 2, 6, and 9. Exogenous *Oct4* showed a signal starting on day 2 (Figure 2c). The exogenous *Oct4* signal peaked on day 9 (Figure 2c). Levels of the retroviral signal followed *Ds-Red* levels (Figure 1). Lastly, the histogram log shift comparison among the exogenous *Oct4* and endogenous *Oct4* on days 2, 6, and 9 showed changes only on the exogenous *Oct4* (Figure 2d). There was no endogenous *Oct4* signal compared to the exogenous *Oct4* signal starting on day 2.

### 3.2. Evaluation of Chondrogenic Trans-Differentiation

**Figure 3:**
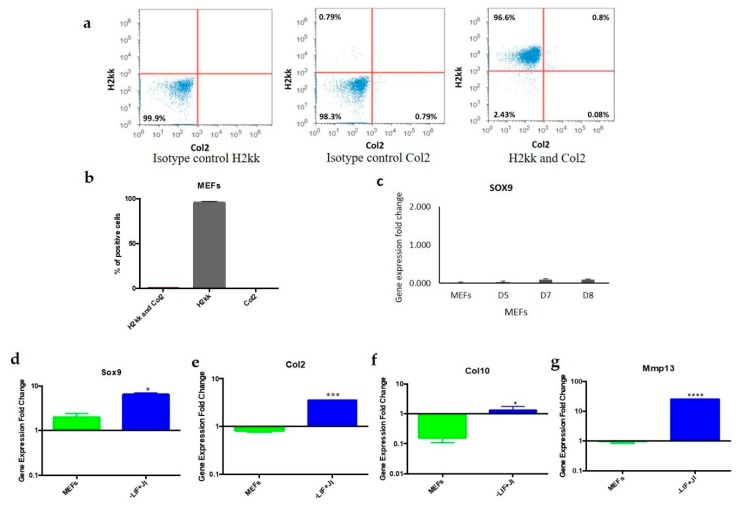
A negligible number of chondroblasts present in the starting MEFs population. 2-D histogram plotted with fluorescent intensity from fibroblast (MEFs) marked with *H2kk* (APC) and *Col2* (FITC) antibodies analyzed by flow cytometry. (**a**) The horizontal-vertical lines divide the cells into four quadrants, which represent cells that were not marked or did not have an expression of those proteins (bottom left), cells with *H2kk* expression (top left), cells with *Col2* expression (bottom right) and cells with expression of both proteins (top right). (**b**) In total, 96.6% of the MEFs population stained positive for *H2kk*, whereas 0.8% positive for both *H2kk* and *Col2*. (**c**) Expression of *Sox9* in MEFs cultured with chondrogenic media during different time points is comparable to control MEFs. (**d**–**g**) Chondroblast gene expression is different in transdifferentiated cells compared to MEFs. OSKM-transduced MEFs were transdifferentiated (−LIF+JI1) and then analyzed for chondrogenic gene expression (*Sox9, Col2, Col10,* and *Mmp13*) with a comparison to MEFs (starting cell population). Gene expression data normalized to MEFs. The figure shows that the expression of Sox9 was five times higher in transdifferentiated cells than in MEFs, with a significant upregulation. * *p* < 0.050.

Flow cytometry confirmed the absence of chondroblasts in the starting MEFs population against the chondroblast marker *Col2* and fibroblast marker *H2kk*. In addition, 96.6% of the MEFs population was positive for fibroblast marker *H2kk*. In total, 0.8% expressed both marker *Col2* and marker *H2kk* while 0.08% were *Col2* positive (Figure 3a,b). Additionally, to rule out the presence of mesenchymal stem cells in the starting population, MEFs were cultured in chondrogenic media for eight days. Cells were tested for the expression of *Sox 9*. The expression of *Sox 9* at the different time points was comparable to MEFs, which confirmed the absence of a considerable number of mesenchymal stem cells in the starting population (Figure 3c). To confirm these results, we compared gene expression for *Sox9, Col2, Col10*, and *Mmp13*, in MEFs to the transdifferentiated chondrogenic aggregates on day 36. The tested genes showed downregulation in MEFs compared to trans-differentiated chondroblast aggregates (Figure 3d–g). *Sox9, Col2, Col10*, and *Mmp13* showed significant upregulation in the transdifferentiated aggregates including *Sox2* (*p* = 0.0131), Col2 (*p* = 0.0008,), *Col1*0 (*p* = 0.0106), and *Mmp13* (*p* = <0.0001).

These results ruled out the possibility that the starting MEF population contained a significant number of mature chondroblasts that could have demonstrated false-positive results in the trans-differentiation protocol.

### 3.3. Characterization of the Kinetics of Trans-Differentiation to Chondroblasts

**Figure 4:**
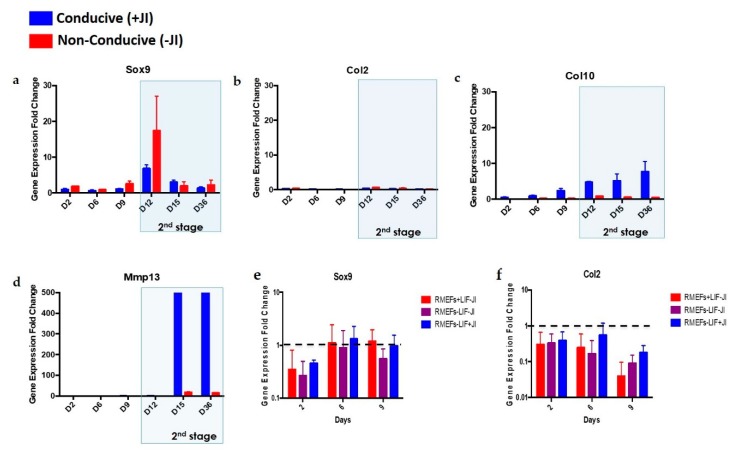
Effect of JI1 to the chondrogenic gene expression kinetics of transdifferentiated aggregates. Cells were transdifferentiated (day 2 to 36) under a conducive and non-conduce media. (**a**) *Sox9* expression demonstrated its upregulation on day 12 (2nd stage) with later gene expression reduction toward the end of the process. Levels of expression were similar for both conditions. (**b**) *Col2* expression was not upregulated in either condition at any time in the process. (**c**) *Col10* was expressed at a higher level in cells under conducive media and expression levels increased with time. No *Col10* expression under non-conducive media. (**d**) The *Mmp13* gene was upregulated under conducive media on day 15, and this continued up to day 36. The *Mmp13* gene was not present in non-conducive media. (**e**,**f**) MEFs do not undergo spontaneous chondrogenesis during the first stage of trans-differentiation either in the presence or absence of LIF or JI1. OSKM-transduced MEFs were cultured in three different conditions (−LIF/+JI1, −LIF/−JI1 and +LIFJI1) and samples on days 2, 6, and 9 were analyzed for *Sox9* and *Col2* gene expression. Both *Sox9* and *Col2* genes showed insignificant upregulation at all time points and conditions.

To understand the kinetics of the trans-differentiation process, we collected cell samples on days 2, 6, 9, 12, 15, and 36 of trans-differentiation. Gene expression fold change of chondroblast markers *Sox9, Col2, Col10*, and *Mmp13* relative to mouse embryonic fibroblast (MEFs) was assessed for each time point. On days 2 and 6 of reprogramming, *Sox9, Col2, Col10*, and *Mmp13* gene expression showed downregulation (Figure 4). On day 9, there was an increase in *Col10* gene expression. Upon exposure to chondrogenic media, *Sox9* (Figure 4a), *Col10* (Figure 4c), and *Mmp13* (Figure 4d) showed upregulation on day 12. *Col2* (Figure 4a) remained downregulated even in the presence of chondrogenic factors. On day 15, there was a reduction in the expression of *Sox9* and *Col10* and a concomitant increase in the expression of *Mmp13*. Lastly, by day 36, the profile was maintained with a slight reduction in the expression of *Sox9*. The expression of the *Col10* and *Mmp13* genes remained similar to our observation on day 15.

### 3.4. Effect of JAK Inhibition on Chondrogenic Differentiation

To investigate the effect of JI1 during chondrogenesis, we compared trans-differentiated MEFs in conducive media (+JI1) to trans-differentiated MEFs in non-conducive media (−JI1). In this case, cells in the absence of JI1 showed that *Sox9* and *Col2* maintained the same pattern as cells in the presence of JI1 (Figure 4a,b), whereas *Col10* was downregulated in non-conducive conditions (Figure 4c). *Mmp13* showed less expression under no JI1 conditions, but, at the same time, was the only upregulated gene (Figure 4d). Overall, *Mmp13* was the only gene upregulated in the absence of JI1 by day 15.

We ruled out spontaneous chondrogenic differentiation of MEFs upon transduction. Three groups of transduced MEFs: +LIF/–JI1, LIF/+JI1, and −LIF/−JI1 were grown for 9 days. We tested samples on days 2, 6, and 9 for the expression of *Sox9* and *Col2*. Results indicated insignificant differences in *Sox9* or *Col2* gene expression (Figure 4e,f).

**Figure 5:**
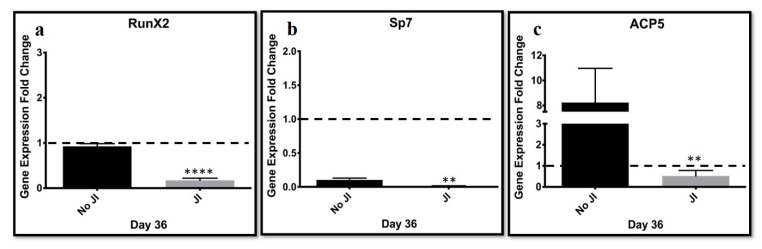
Bone markers gene expression is stimulated in the absence of JI1 during trans-differentiation. (**a**,**b**) Downregulation of *Runx2* and *Sp7* in the presence of JI1. (**c**) The marker *ACP5* showed the highest expression (7-fold) in non-conducive media compared to the conducive conditions. ** *p* < 0.010.

We evaluated the potential for bone formation by measuring the gene expression of bone markers. As a control, MEFs that were trans-differentiated in nonconductive media (−LIF−JI1) underwent the same trans-differentiation process. We analyzed the gene expression fold change of the bone markers *Runx2, Sp7*, and *ACP5* relative to mouse embryonic fibroblasts (MEFs) (Figure 5). *Runx2* (*p* = 0.0001), *Sp7* (*p* = 0.0048), and *ACP5* (*p* = 0.0083) gene expressions were significantly low compared to the non-conducive (−JI1) condition. These results suggest that the presence of JI1 increases the efficiency of trans-differentiating MEFs into cartilage by avoiding bone formation.

### 3.5. Detection of Hyaline and Hypertrophic Cartilage in Subcutaneous Aggregate Transplants

**Figure 6:**
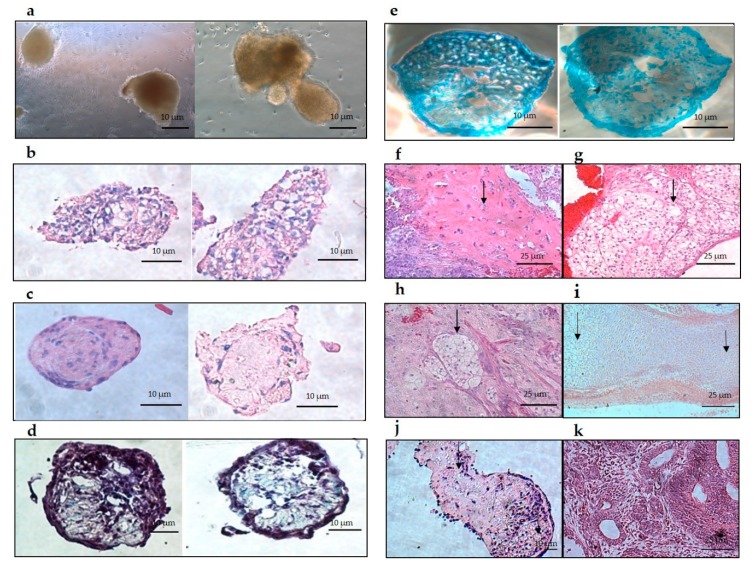
Cartilage structure and glycosaminoglycan (GAG) are present in the transdifferentiated aggregates. (**a**) Compact cartilage aggregates in culture. (**b**) Cartilage-like tissue stained by H & E. (**c**) Hyaline cartilage-like tissue stained by H & E with sparse chondroblasts in the lacunae structure (Scale bar 10 μm). (**d**) (GAG) deposition stained by Safranin-O. (**e**) (GAG) deposition stained by Alcian blue. (**f**) H & E staining of hyaline chondroblasts and hyaline matrix found in vivo following transplantation in mice. (**g**,**h**) H & E staining of hypertrophic chondroblasts in a subcutaneous capsule. (**i**) H & E staining of developing limb bud in a mouse embryo showing natural, normal hyaline (right arrow) and hypertrophic (left arrow) chondroblasts (Scale bar 25μm). (**j**) H & E staining of chondroblast aggregates differentiated from mouse iPSCs showing hyaline cartilage-like tissue with chondroblasts in lacunae (left arrow) and hypertrophic chondroblasts (right arrow) (Scale bar 10 μm). (**k**) IPSCs derived teratoma showing heterogenous tissue formation (Scale bar 25 μm).

H & E staining of the resulting aggregates showed clear hyaline and hypertrophic cartilage (Figure 6b,c) the differentiated chondroblasts and cartilage-like tissue resembled chondroblasts differentiated from mouse iPSCs aggregates (Figure 6j) and chondroblast found in developing mouse embryos (Figure 6i). Alcian blue and Safranin-O demonstrated the presence of glycosaminoglycans (GAG), which confirmed cartilage formation (Figure 6d,e). Subcutaneous transplantation of day 36 chondroblast aggregates into the inner thigh of SCID mice resulted in cartilaginous tissue. Three weeks after transplantation, the histologic analysis showed cartilage structure formation. Lacunae structures of hyaline (Figure 6f) and hypertrophic cartilage (Figure 6g,h) formed. We were unable to detect any other type of tissue using H &E compared to iPSCs derived teratomas where iPSCs were transplanted in subcutaneous capsules in mice and left for spontaneous differentiation to take place. Heterogeneous differentiation and different types of tissues were formed (Figure 6k).

## 4. Discussion

**Figure 7:**
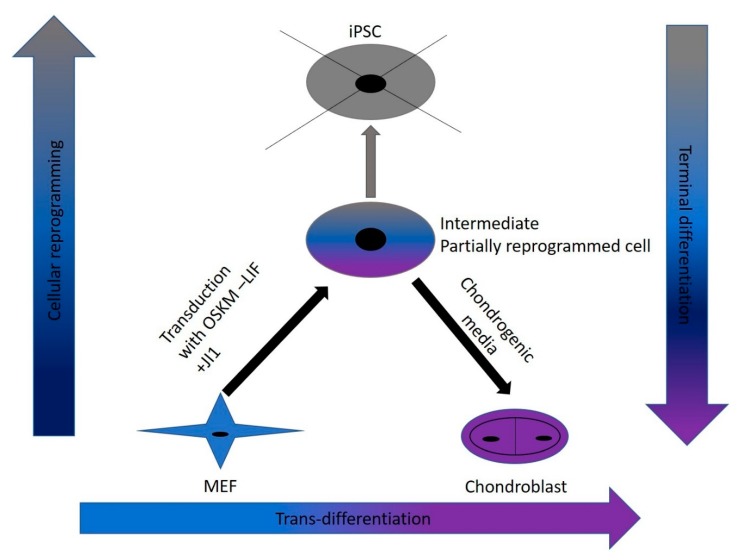
Schematic of the methodology used in this study. Retroviral transduction partially reprogramed MEFs in the absence of LIF and the addition of JI1. This approach put the cells into a plastic state where the chromatin is open enough for the chemicals and small molecules in the chondrogenic media to reprogram cells to chondroblasts without reaching the iPSCs state directly.

Trans-differentiation is a reprogramming process that uses lineage-specific transcription factors as a potentially faster and tumor-free alternative to iPSCs [7]. In this study, we aimed to find a safe and rapid method for generating chondroblasts. We performed the transduction of mouse embryonic fibroblasts (MEFs) with the well-known OSKM factors in the absence of LIF and with inhibition of the JAK-STAT pathway using the JAK inhibitor (JI1). This approach arrested iPSCs formation. It allowed for the partial opening of the chromatin enough for the second stage of the trans-differentiation process [17]. In the second stage, we exposed the cells to chondroblast differentiation media in the presence of growth factors (*TGF-β* and *BMP2*) that gave the cues for the cells to turn into chondroblasts for cartilage formation (Figure 7).

This relative fast approach could lead to a near-term clinical treatment that utilizes the patients’ fibroblasts to create chondroblasts and treat osteoarthritis, especially with the selection of the optimum scaffolds and implantation conditions [25].

**Figure 8:**
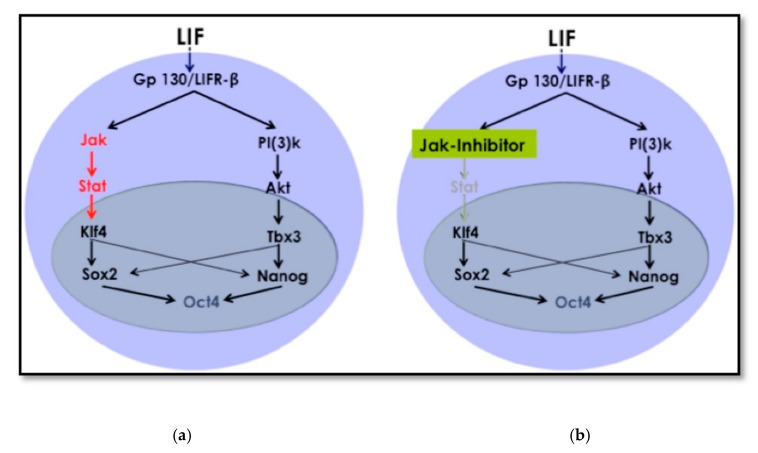
Schematics of the LIF/JAK-STAT3 pathway active and blocked by a JAK inhibitor. (**a**) LIF binds to its Gp130/LIFR-β and activates the JAK-STAT3 pathway. Its activation leads to the direct stimulation of the pluripotency circuitry transcription factors in the nucleus for pluripotency reprogramming. (**b**) When JAK-STAT and no other pathway is inactivated, the stimulation of the pluripotency circuitry decays, and the reprogramming process is affected.

Cellular reprogramming using the OSKM factors is a well-known and established method [16]. The mechanism and kinetics of the reprogramming process to iPSCs are known, and this includes cell signaling pathways that crosstalk during the process until cells acquire pluripotency. The LIF/JAK-STAT3 pathway is one of the critical signaling pathways for maintaining pluripotency [26]. In embryonic stem cells (ESCs,) LIF targets different signaling pathways in addition to the JAK-STAT3, among which is phosphatidylinositol 3-kinase (PI3K). This is also known as Akt and extracellular signal-regulated kinases (Erk). The signaling cascade leads to the activation of the core circuitry of pluripotency transcription factors *Oct3/4, Sox2*, and *Nanog.* LIF was deemed to directly contribute to the core circuitry of pluripotency (Figure 8) [27].

LIF first reaches the receptor gp130 and sends a signal to JAK, which later phosphorylates TSTAT3 to activate Klf4 (Figure 8). Klf4 will then activate *Sox2*, which, in sequence, activates *Oct3/4*. Therefore, this promotes pluripotency. The protocol we used depending on removing LIF and inhibiting JAK. This modification to the traditional retroviral transduction protocol initiates the reprogramming process. The chromatin becomes loose enough only for chemicals and small molecules that support differentiation to direct cell fate towards the desired cell type.

This trans-differentiation approach was executed by Efe and colleagues to generate cardiomyocytes by MEFs trans-differentiation with OSKM factors [17]. We successfully recapitulated this trans-differentiation protocol and used a chemical differentiation cocktail for cardiac differentiation that we established in our lab. The results showed beating cells 10 days faster than generating cardiomyocytes from iPSCs. The presence of the markers *ANF*, *αMHC*, and *NKX2.5* confirmed the effective trans-differentiation. Among the cardiac markers that Efe et al. analyzed [17], they also assessed *NKX2.5*, which is a transcription factor that regulates heart development, and found that *NKX2.5* was upregulated on day 9.

In contrast, in this preliminary study, we found that *NKX2.5* was upregulated later by day 11. The cardiac media used for this initial study might have delayed the upregulation of the *NKX2.5* marker given that cells react differently in response to the composition of the differentiation media. Similar to our results, they obtained higher efficiency in cardiomyocyte formation compared to cardiac differentiation from iPSCs [17] (Data not shown). The same trans-differentiation approach was used in this study for the first time to generate chondroblasts. The chondroblast differentiation media used in this study was developed in our lab [28] and was reported to create chondrogenic gene expression.

This study also shows that chondroblast differentiation media with micro-mass formation was more effective in this trans-differentiation approach than with ESC-chondroblast differentiation. For the second stage (day 10), cells were plated in 10 μL micro-mass cultures, according to Yamashita and collaborator’s protocol. At this time, it was not clear whether the pre-iPSCs generated in stage 1 of this trans-differentiation protocol would be able to form micro-masses. Yamashita et al. [28] reported that, when differentiating ESCs into chondroblasts using this approach, discordant cells die by apoptosis, undergoing a selection process where only cells that will turn into those of the chondrogenic lineages survive. We observed that forming micro-mass did not compromise the MEF-chondroblast trans-differentiation. Additionally, in contrast to Yamashita et al. results [28], cells successfully formed cartilage aggregates and did not separate from the micro-mass and die. This observation suggested that using a micro-mass approach in trans-differentiation for chondroblast derivation is more efficient than deriving chondroblasts from ESC differentiation via micro-mass culture [28]. The presence of chondrogenic markers in trans-differentiated aggregates confirmed the successful chondroblast formation. The markers *Sox9, Col2, Col10*, and *Mmp13* are specific markers for the different stages of chondroblast differentiation.

*Sox9* is a transcription factor that orchestrates the expression of collagen genes such as *Col2* and *Col10. Col2*, along with others, form the cartilage-specific collagens that will later serve as a scaffold for mineralization. Next, the proliferating chondroblasts express the *Col10* gene, among other markers. *Mmp13* participates at the end of the process by degrading the cartilage matrix for mineralization to be initiated [29,30]. Our results showed increased expression of *Sox9, Col10*, and *Mmp13* at the early stages of chondrogenesis. These markers maintained their upregulation up to day 12, where they became downregulated except for the levels of *Mmp13* gene, which remained high.

Following this assessment, on day 40, Yamashita et al. observed the expression of these same markers. This gene expression pattern indicated the formation of hypertrophic chondroblasts for later bone formation, given that *Mmp13* was upregulated. Our results showed faster cartilage aggregate formation compared to ESCs differentiation to chondroblasts using micro-mass composition [28].

Histology results demonstrated positive staining to a cartilage-like structure for hyaline and hypertrophic cartilage, with no other type of tissue detected throughout the aggregates. Trans-differentiated aggregates showed a similar structure to aggregates at different stages of ESC chondrogenic differentiation [31]. These findings suggest that trans-differentiation develops the same results as ESC-cartilage differentiation [32]. Additionally, trans-differentiated aggregates clearly showed glycosaminoglycan (GAGs) deposition when stained with Alcian blue or Safranin-O. Upon transplantation of trans-differentiating aggregates into SCID mice, only hyaline and hypertrophic cartilage developed. These structures are consistent with the structures found in the in vitro sectioned aggregates.

MEFs are a heterogeneous population that might include other types of cells, such as chondroblasts and mesenchymal stem cells. Flow cytometry results indicated that the starting MEF population was uniform and did not include a significant number of chondroblasts. A total of 96% of MEFs expressed the fibroblast marker *H2kk* only. In addition, 0.8% of the population expressed both *H2kk* and the chondrogenic marker *Col2*. Chondroblast survival was minimal since the media used did not contain the proper growth factors to promote chondroblast cell division and survival. MEFs were cultured in chondrogenic media to exclude the presence of mesenchymal stem cells that could differentiate to chondroblasts. The results showed gene expression that closely resembled MEFs, which showed no expression of *Sox9*. On the other hand, if chondrogenic or residual cells would survive in the MEFs population, once plated for trans-differentiation, they are not likely to be reprogrammed with the OSKM factors since the target cells should be actively dividing. This criterion does not apply on chondrocytes due to quiescence [33].

We used *Oct4* expression together with *Nanog* and *Rex1* expression to exclude intermediate iPSCs formation. Flow cytometry results for the first stage of trans-differentiation showed an exogenous *Oct4* signal and no endogenous *Oct4* signal, which supports the absence of iPSCs formation since endogenous *Oct4* showed no expression during this stage of trans-differentiation. Per this finding, the expression of *Nanog* and *Rex1* genes in the transdifferentiated MEFs was minimal when compared to iPSCs.

JI1 enhances trans-differentiation efficiency in cardiomyocytes and neural progenitor cells [22]. However, it has never been reported for chondrogenic trans-differentiation. In this study, we compared the gene expression profile of *Sox9, Col2, Col10*, and *Mmp13* in cells that were trans-differentiated in conducive media (+JI1) to trans-differentiated cells in non-conducive media (-JI1). Interestingly, *Sox9* presented a similar pattern in both conditions. On the other side, *Col10* showed upregulation in conducive media and no expression in non-conducive media. Similarly, *Mmp13* expression was higher in conducive media compared to the non-conducive media (−JI1). Overall, under conducive media, *Sox9, Col10,* and *Mmp13* were upregulated, whereas, in non-conducive media, there was only a small amount of expression of the *Mmp13* gene. *Mmp13* is involved in the degradation specifically of collagen type II, as it is overexpressed in OA cartilage tissue [34]. In this scenario, it might be that trans-differentiation without JI1 leads to an imperfect cartilage structure. This finding, in consequence, highlights how trans-differentiation efficiency increases upon the usage of JI1.

Our results showed the absence of tumor formation upon in vitro transplantation. These findings could be due to the role of JI1 in preventing iPSCs formation. iPSCs (and ESCs) have the potential to form teratomas when transplanted. Tumor formation is a significant limitation slowing the clinical application of pluripotent stem cells [35,36]. This trans-differentiation approach can overcome this problem.

Trans-differentiation of MEFs formed mature chondroblasts by day 15 (Figure 4), which is half the time required for reprogramming MEFs into iPSCs and then differentiating them into chondroblasts. We defined mature chondroblast formation, according to Yamashita et al. [31]. Our results displayed the expression of *Col10* and *Mmp13* genes by day 15, which likely indicates hypertrophic chondrocyte formation. It has been reported that reprogramming experiments with iPSCs formation take a long time for their first appearance in culture [37]. In comparison to trans-differentiation, the same time frame is needed for trans-differentiated MEFs to become hypertrophic chondrocytes. In another study, turning osteoarthritic chondrocytes into iPSCs and then differentiating them into healthy chondroblasts took, on average, 34 days [38]. This period is double the time required for obtaining chondroblasts via trans-differentiation. Overall, our results suggest that the trans-differentiation of MEFs into the chondrogenic lineage using OSKM factors is faster than conventional methods of using iPSCs.

JAK inhibition might have blocked bone formation. Bone markers *Runx2*, which determines osteoblast lineage, and *Sp7* direct the fate of cells to osteocytes by blocking their differentiation into chondroblasts and *ACP5*, which is an acid phosphatase 5. A tartrate-resistant protein was assessed. The results were compared to transdifferentiated cells cultured in the absence of a JI1 or non-conducive condition. *ACP5* highly expressed in the lack of JI1 was compared to trans-differentiated cells in the presence of JI1. *Sp7* and *Runx2* genes were expressed slightly higher in the non-conducive (−JI1) condition when compared to the condition with JI1. These results suggest that JI1 leads to more efficient trans-differentiation into chondroblasts. JI1 keeps the cells from continuing to bone formation. Thus, there is the potential to tailor this trans-differentiation approach to form only hyaline cartilage, perhaps by adjusting the JI1 exposure time. 

Other approaches to obtain hyaline cartilage can also be applied. For example, as previously mentioned, *Sox9,* while overexpressed in the chondroblasts of the mouse embryos, results in delayed hypertrophic chondrocyte differentiation [39]. Thus, the manipulation of *Sox9* gene expression might be an excellent pathway to direct trans-differentiation to a more hyaline cartilage fate.

Our results suggest that this trans-differentiation method is more inclined toward the formation of hypertrophic chondrocytes for consequent bone development [40]. We observed an early upregulation of *Col10* on day 6, as well as a later upregulation of *Mmp13* on day 12, which mimics a hypertrophic cartilage gene expression profile. Although endochondral ossification does not occur in vitro, it could happen in vivo upon transplantation. Yamashita and colleagues observed this when ESCs were differentiated to chondrocytes in micro-mass culture [31]. Hence, more extended periods of incubation following transplantation would have also allowed us to better study whether cartilage trans-differentiation could lead to endochondral ossification following transplantation.

Trans-differentiation offers the potential to find the window for hyaline cartilage formation with the advantage that it could be tumor-free. A good example is that this window has been seen before in another chondroblast trans-differentiation approach. Hiramatsu et al. 2011 [21] were able to find this window in their protocol for hyaline cartilage formation by retaining the expression of *Sox9*. This transcription factor was reported to delay hypertrophy [25,39]. In their work, they used viral particle transduction together with the doxycycline inducibility of the transgenes. Hence, when *Sox9* maintained its expression, cells remained in a hyaline cartilage-like state. In contrast, when *Sox9* didn’t continue expression, cells moved into hypertrophy.

## 5. Conclusions

Altogether, the results discussed above demonstrated the following.

- Transduction of MEFs with OSKM factors with the removal of LIF and addition of JI1 put the cells in a plastic state where the chromatin was open enough for chemicals and small molecules responsible for chondrogenesis to direct the cells from the fibroblast fate to the chondroblast fate.
- There was no intermediate iPSCs formation. Moreover, a negligible number of mesenchymal stem cells and mature chondroblasts was present in the starting population.
- The time needed for trans-differentiation is less than half the time required for the differentiation of chondroblasts from embryonic or induced stem cells (15 days for trans-differentiation versus 34 days for chondrocyte differentiation from ESC or iPSCs).
- This approach can lead to a near personalized clinical treatment that will allow less morbidity for OA patients.
- This approach will also eliminate the potential of graft rejections and tumor formation after transplantation.
